# Minimally invasive approaches for the correction of adult spinal deformity

**DOI:** 10.1007/s00586-012-2344-6

**Published:** 2012-05-10

**Authors:** Neel Anand, Eli M. Baron

**Affiliations:** Cedars Sinai Spine Center, Cedars Sinai Medical Center, 444 S. San Vicente Blvd., Suite 800, Los Angeles, CA 90048 USA

**Keywords:** Adult deformity, Minimally invasive

## Abstract

**Introduction:**

Spinal deformity surgery is historically associated with significant blood loss and medical complications. Minimally invasive deformity correction is a promising approach to spinal deformity surgery where deformity correction and fusion can be achieved with less tissue trauma, reduced blood loss and potentially less complications.

**Materials and methods:**

We discuss technical aspects of minimally invasive deformity correction, review the transpsoas and presacral approaches for discectomy and fusion, and review multilevel posterior percutaneous pedicle instrumentation and rod placement for deformity correction. We also review our results using these techniques and review the literature regarding outcomes in this emerging area of spinal surgery.

**Conclusions:**

Minimally invasive deformity correction is a promising method of spinal deformity correction. Early clinical results are similar to open techniques, with reduced blood loss and less complications than traditional approaches. Meticulous technique and careful patient selection are required for good results and to avoid complications.

## Introduction

Minimally invasive deformity correction and fusion remains an exciting field of spine surgery. Traditionally, adult deformity surgery is associated with high-volume blood loss and significant medical complications [1–3]. Additionally, given the fact that much of the adult deformity population is treated for lumbar degenerative scoliosis, disease processes seen in the elderly such as diabetes mellitus and coronary artery disease further add risk for potential medical complications [4]. Given this, a minimally invasive approach to the treatment of adult deformity is particularly attractive. In order for a minimally invasive approach to be widely adapted, it needs to be (1) effective when compared with traditional open approaches, (2) have reasonable operating times with reduced medical complications and reduced blood loss, and (3) must be technically feasible in order to be duplicated and widely adapted. We review our experience with minimally invasive deformity correction and particularly discuss the minimally invasive approaches we have adopted for the correction of adult scoliosis.

### Indications

**Fig. 1:**
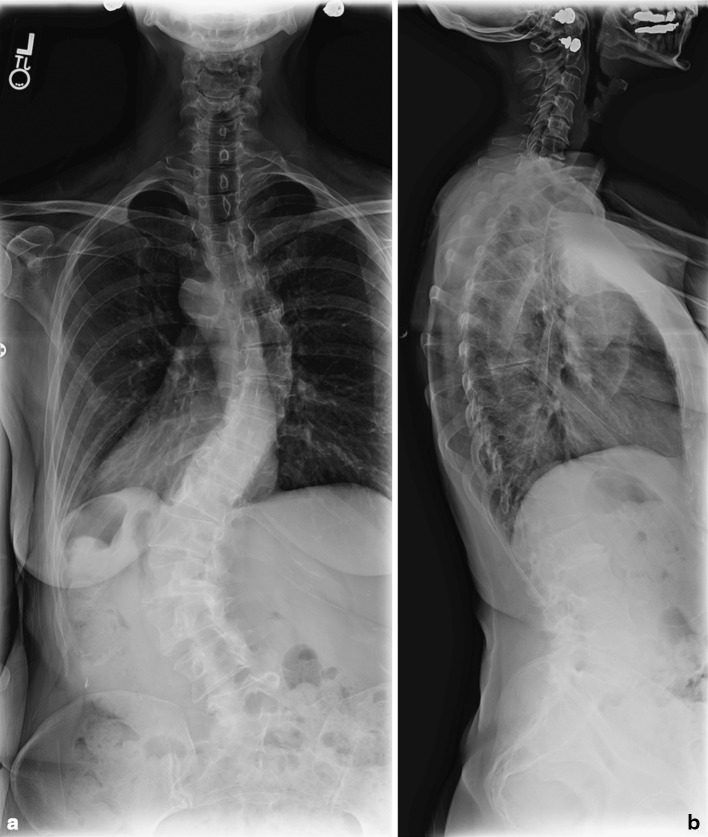
**a**, **b** Scoliosis films in a 53-year-old female with adult idiopathic scoliosis demonstrating a left 55° curve extending from T10–L4 with lumbosacral obliquity. The patient also has a 34-degree compensatory curve extending from T5 to T9. The patient presented clinically with progression of her deformity and increased pain

Patients who undergo minimally invasive correction and fusion for adult spinal deformity are typically treated for symptomatic back and leg pain. This includes adult idiopathic scoliosis, iatrogenic scoliosis and lumbar degenerative scoliosis. Patients typically exhaust numerous conservative therapies, including physical therapy, epidural and facet injections, and other conservative measures prior to being considered for surgery. Our primary indication for correction of adult spinal deformity remains mechanical low back pain. This pain is characterized by stiffness in the morning with progressively increasing pain with activity and worsening pain as the day goes on. This may or may not be accompanied by radiculopathy with claudication. Other indications have been proposed for surgery for adults with scoliosis, including curve progression, sagittal and/or coronal imbalance with unremitting back pain, curve flexibility, curve of greater than 50° when decompression is considered, documented history of progressive curve, radiculopathy on the side of the concavity of the curve due to foraminal stenosis, lumbar hyperlordosis, patients with a history of flat-back syndrome and back pain, fixed lateral listhesis within the degenerative curve when motion is present on side-bending films and when extensive decompression including facetectomy or the violation of the pars is planned [5]. All patients are worked up with 36-in. standing films (Fig. 1). We also obtain an MRI in most patients to ascertain the quality of the lumbosacral disc as well as the most proximal normal disc. We instrument all levels within the Cobb angle. If the fusion crosses the thoracolumbar junction, we stop the proximal level at the first normal parallel disc irrespective of whether it is at L1, T12 or T11. We also obtain a CT scan if there is any concern that there is a true fusion of the spinal segments. A bone density scan is performed on all patients greater than 50 years and we would caution against minimally invasive correction when the *T* score is less than 2.0. We also obtain an MRI of the sacrum if the AxiaLIF technique is used to fixate the L5–S1 segment. This helps evaluate the presacral space for any adhesions as well as rule out aberrant vasculature that crosses the midline on the anterior surface of the sacrum.

As of today, we would recommend against the application of minimally invasive techniques for spinal deformity in curves with a Cobb angle greater than 90°, sagittal imbalance greater than 10 cm, rigid kyphotic deformities, deformities with fused spinal segments and osteoporosis with *T* score of less than −2.0. We usually use open traditional methods to facilitate performance of osteotomies. We also would not recommend these techniques in high-grade spondylolisthesis. A relative indication is progressively worsening deformity with pain as the rib cage abuts the pelvis. Numerous factors come into play if surgery is considered. Medical co-morbidities need to be carefully evaluated, as with any major surgery, and osteoporosis carefully screened. Surgical planning should take into consideration the type, extent and magnitude of the curve being treated. Curve flexibility should be carefully evaluated and truly rigid curves as evidenced by fusion of the spinal segments are not candidates for minimally invasive techniques. Both flexible and stiff curves can be treated minimally invasively.

### Technical considerations

Our minimally invasive strategy has mainly relied on three techniques for the correction of spinal deformity and fusion. These include the transpsoas approach for discectomy, release and interbody fusion, the presacral approach for fixation and interbody fusion at L5–S1 and sometimes L4–L5, and multilevel percutaneous pedicle screw and rod placement. In carefully selected patients as mentioned above, we have not found the need to perform osteotomies to obtain sagittal or coronal balance. We describe the three techniques below and then review outcomes using these techniques.

### Transpsoas discectomy and fusion

Transpsoas discectomy and fusion builds upon earlier experiences where lateral interbody fusions were performed using laparoscopic techniques using BAK cages [6, 7]. Numerous papers have discussed the modern techniques of transpsoas lateral fusion [8–10].

We prefer to use a regular radiolucent sliding table with a kidney rest to position the patient. The patient is placed in the right lateral decubitus position with the left side up in all cases. This minimizes the risk to the vascular structures [11], especially at L4–5. The patient’s iliac crests are positioned just below the level of the kidney rest and the kidney rest is elevated maximally to maximize the distance between the iliac crest caudally and the rib cage rostrally. An axillary roll is placed. Additionally, we secure the patient with bolsters and strapping tape to the table. We use towels between the patient’s skin and the strapping tape to avoid skin abrasions. The top hip is flexed to relax the psoas muscle. We then place further padding around the fibula to minimize risk of peroneal nerve palsy and further secure the patient’s top leg to the table using strapping tape.

After we position the patient, a C-arm is brought in using lateral projection and is used to plan incisions to access the appropriate disc spaces. We target the junction of the anterior and middle third of the disc space to minimize neurologic risk, as per Moro et al. [12]. Afterward, oblique incisions are planned running with the grain of the external abdominal oblique musculature. We typically use one incision for L4–L5 directly over the disc space. Then we try to do two levels through one incision by marking the skin in between, but the discs are accessed through separate fascial incisions so as to have direct access to the disc space in question.

A scalpel is used to incise the skin. Subcutaneous bleeders are gently coagulated with a Bovie. Access to the retroperitoneal space is obtained through the incision marked for the L4–5 space. The surgeon’s gloved finger is directed toward the iliac crest and then the soft tissue is swept along the top of the crest to the inside of the crest at the Petit’s triangle. The retroperitoneal space is entered in this fashion and the smooth surface of the inside of the iliac crest is swept posterior to the anterior iliac, persuading the peritoneum and its contents anteriorly. The finger is then turned cephalad to palpate the transverse process and undersurface of the 12th rib, confirming clearance of the retroperitoneal space. The transverse process can also be palpated for further confirmation.

Under fluoroscopic guidance, a PAK (percutaneous access kit) needle is escorted to the level of the relevant disc. The junction of the anterior and middle third of the disc space is targeted. The PAK needle is then advanced into the disc and a guide wire is placed. Its location is confirmed under lateral fluoroscopy. This image is saved. Under AP fluoroscopic guidance, the PAK needle is removed and serial dilators are placed over the guide wire. We monitor the patient with continuous free-running EMG at all times. If the free-running EMG fires a signal, we stop and reassess or redirect. If the signals remain persistent, the needle is not taken through the psoas, but triggered EMG is also used; if that is positive too, then “shallow docking” is performed. This consists of placing the dilators above the psoas and all dissection through the psoas is done under direct vision with the help of free-running EMG. If the lumbar plexus is encountered during this dissection posteriorly, then we can redirect anteriorly. If the nerve is directly in the path of the interbody placement, we choose to abandon the lateral transpsoas approach, as we feel that dissecting and mobilizing the nerve are not safe through a small tubular corridor and only lead to further stretching and nerve injury. When the free run EMG has been normal throughout, we have never encountered the nerve and have had no neurological issues. Free run and triggered EMG is also used when placing dilators. Following the final dilator placement, an appropriate size, expanding tubular retractor is placed. This is secured typically with a retention pin. Our experience has been largely with the Medtronic Quadrant Retractor, which is readily docked with a pin and secured with an articulating arm (Medtronic Sofamor Danek, Memphis, TN).

**Fig. 2:**
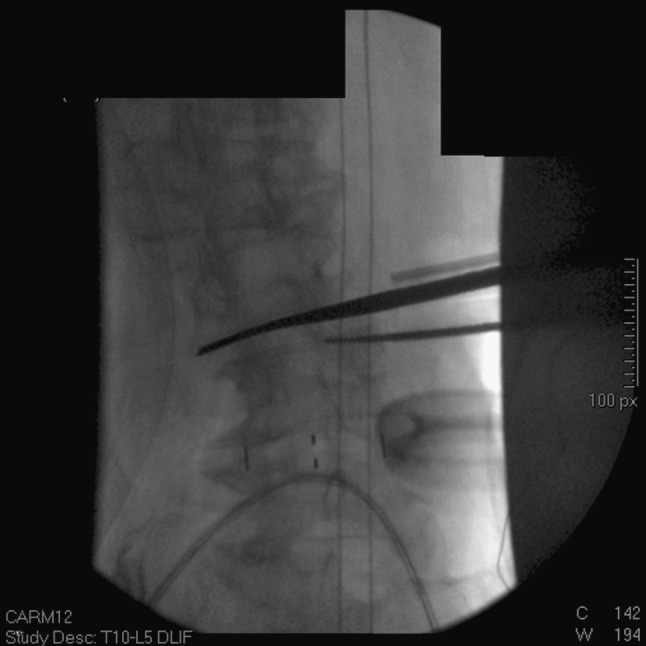
AP fluoroscopic image demonstrating a Cobb elevator used for discectomy at L3–4. Note the docked tubular retractor

The guide wire is left in place, the light source is attached and the area under the tube visualized. A stimulating probe with triggered EMG is used to check the area visualized. The guide wire is used as a guide to perform the annulotomy and the reference from the lateral and AP fluoroscopy is very useful. We use a 15-blade to cut the disc. A Cobb elevator is then used to separate cartilaginous from bony endplate (Fig. 2). A series of rasps, rongeurs and curettes are used to radically excise the disc. Great care is taken not to violate the endplates. Serial trials are then used and, once an adequate size is determined, a polyetheretherketone spacer (PEEK) is packed with rhBMP-2/ACS (INFUSE, Medtronic Sofamor Danek, Memphis, TN) and Grafton Putty Demineralized Bone Matrix (DBM) (Osteotech, Eatontown, New Jersey). In terms of dosing of the rhBMP-2, we use 2–4 mg in each PEEK cage [9]. We use lordotic spacers at every level. We choose the best-fitting trial and restore disc height. Too large a trial may damage the endplates. Additionally, great care must be taken not to violate the anterior longitudinal ligament, as it can result in difficulty in retaining the spacer in the space as well as possible injury to the visceral structures anterior to the spine. The spacer is filled with DBM and rhBMP-2 and impacted into the disc space under fluoroscopic control. The C-arm is used to confirm spacer placement, both in the AP and lateral planes, before the insertion handle is removed, so that any adjustments can be made as needed. The retractor pin followed by the retractor is removed under visualization to confirm lack of bleeding. The technique is then repeated for additional levels as needed.

This procedure is done from caudal to rostral. When approaching, the thoracolumbar junction, however, the diaphragm may need to be pushed upward, especially at T12–L1. At T11–12, the entry is usually in the thoracic cavity and the diaphragm is pushed downward with a gloved finger while docking the retractor. We also have the anesthesiologist hold the expiration to keep the lung away from the surgical field while passing the PAK needle and docking into the disc space. We have not found the need to use a chest tube; rather upon closure, a red rubber catheter is used to aspirate any air. A watertight closure is then performed around the red rubber catheter as it is withdrawn. Simultaneously, suction is performed while having the anesthesiologist have the patient perform a Valsalva maneuver. A purse-string suture is cinched down while the suction maneuver is performed and the red rubber tube is withdrawn. We have not had to use chest tubes, as typically postoperative chest X-rays have shown mild pneumothorax of less than 10 %.

**Fig. 3:**
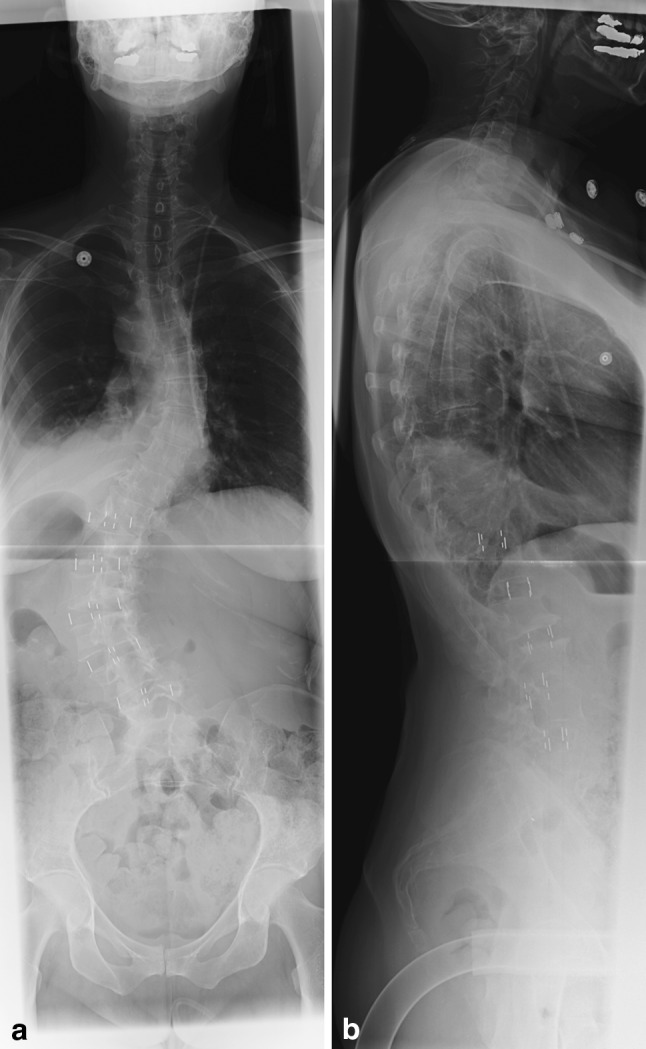
**a**, **b** The 36-inch standing films post transpsoas (lateral) discectomy and interbody fusion from T12–L5. The thoracolumbar curve now measures 50° and the thoracic curve measures 34°. Note how minimal deformity correction was achieved after transpsoas discectomy and interbody fusion

Usually, we wait 2–3 days to stage the posterior portion of the surgery, which includes the presacral approach for interbody fusion and also posterior spinal fusion with deformity correction. This allows us to see if any radicular symptomatology has resolved by the transpsoas discectomy and interbody fusion. We also obtain 36-inch standing films before the second stage and reassess both coronal and sagittal alignment so as to dial in the appropriate correction (Fig. 3).

### Presacral approach for discectomy and interbody fusion

Typically, we perform the presacral approach for interbody fusion prior to inserting pedicle screws. We will, however, perform pedicle screw insertion with rod insertion, if there is preexisting lumbosacral junction obliquity. If obliquity is present, we will correct it with pedicle screws prior to performing the presacral approach for transsacral discectomy and fusion. The presacral approach for discectomy and interbody fusion was described by Marotta et al. using the corridor first clinically described by Cragg et al. [13, 14]. There has been considerable experience with this procedure with low risk of vascular and viscous injury [15]. Prior to performing this approach, it is imperative to review a preoperative pelvic MRI. This is to rule out an aberrant midline vasculature at the level of S1–S2, which would prohibit performance of this procedure. Additionally, if severe compressive pathology is present or other contraindications to transsacral fusion exist, then transforaminal lumbar interbody fusion (TLIF) or an ALIF performed minimally invasively at L5–S1 would be a consideration.

The patient is positioned prone on a Jackson table. We use padding to elevate the thighs and legs. Additionally, the thighs are kept slightly separated to allow working corridor for the surgeon’s hand. The rectal area is prepped and isolated. The skin of the thoracolumbosacral coccygeal spine is then prepped and draped in the usual manner. Typically, we plan a 1-inch incision by the midline near the sacrococcygeal junction. Sometimes, we will make this incision off the midline, particularly when there are questions regarding the patient’s hygiene. A blunt probe is introduced by the paracoccygeal notch. The probe is popped through the sacrospinous ligament in a controlled fashion and the hand immediately dropped between the legs to hug the anterior surface of the sacrum. Fluoroscopy confirms the position of the probe, which is then marched up the midline along the anterior surface of the sacrum via the presacral corridor. Great care is taken not to deviate into the ventral sacral neuroforamina. This is done using strict biplanar fluoroscopy.

Once the blunt probe reaches the S1–S2 junction and appropriate trajectory across the L5–S1 disc space is ascertained, the blunt introducer is removed from the assembly and a sharp guide pin is malleted into the sacrum. An extension to this guide pin is attached to extend the length of the guide pin. This is followed by a 6-mm dilator followed by an 8-mm cannulated dilator, which are placed over the guide pin. This is also followed by a 10-mm dilator, which has a thin-walled dilator sheath slid over the 8-mm dilator. A cannulated slap hammer is then used to anchor the 10-mm dilator into the sacrum.

Afterward, a 9-mm reamer is used to drill a core of bone out to the L5–S1 disc space. This bone is meticulously saved for autogenous bone graft.

**Fig. 4:**
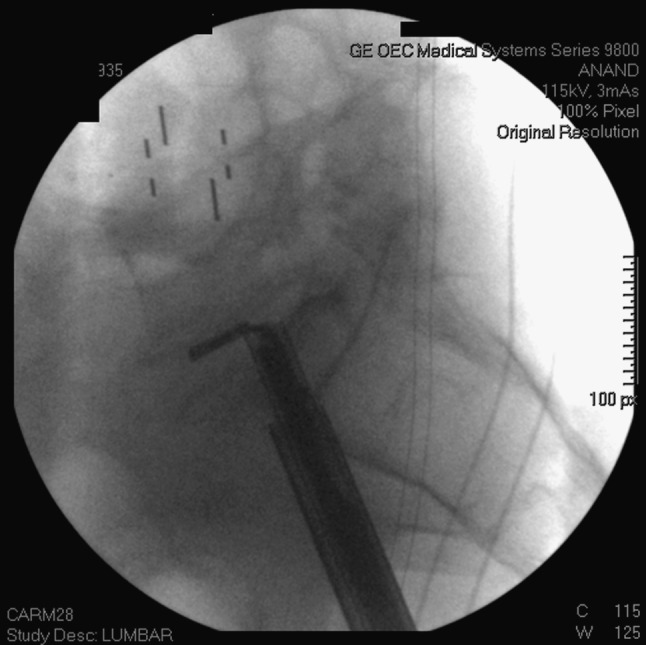
Lateral fluoroscopic image demonstrating Nitinol disc cutter being used in discectomy as part of the MIS L5–S1 discectomy and interbody fusion procedure

Under strict fluoroscopic control, a series of Nitinol cutters, rasps and brush devices are used to radically excise the disc (Fig. 4). The endplates are carefully prepared with removal of disc through multiple passes of alternating nitinol cutters and brushes. The disc space is then irrigated out with bacitracin-containing saline solution. In performing the discectomy, great care is taken to avoid the posterior disc, as this prevents graft material from migrating into the spinal canal.

After irrigation of the disc space, the disc space is grafted with local bone autograft, Grafton Putty DBM and 2.1 mg of rhBMP-2/ACS using the manufacturer-supplied funnels [9]. Subsequently, a smaller twist drill is used to drill into the L5 vertebral body. A guide wire is used to measure the appropriate length of a TranS1 Axial 3D screw. The guide pin is left in position, while the working channel is removed. A larger exchange cannula followed by a larger working channel is placed into position. This is further secured to the ventral surface of the sacrum with a retention wire. Subsequently, a titanium axial 3D screw assembly is placed across the guide wire and screwed across the sacrum, across the L5–S1 disc space into the L5 vertebral body. A universal plug is then placed into its distal end. Similar technique is available for a two-level discectomy and fusion at L4–5 and L5–S1. This is described elsewhere [16].

### Percutaneous pedicle screw and rod placement

Posterior percutaneous pedicle screw and rod placement is critical for the minimally invasive correction of deformity. In fact, it is the rod placement that is often critical to achieving deformity correction. As far as skin incision, one can either use a single vertical incision extending the length of the deformity with individual fascial incisions or use multiple paramedian skin and fascial incisions. In general, these incisions are planned by the lateral border of the pedicles as deemed on AP fluoroscopy.

In either case, the Jamshidi needle enters through either the skin or the fascia by the lateral border of the pedicle. The Jamshidi needle is then advanced down through the soft tissue, approximately on the left side, the 10 o’clock position of the pedicle, or on the right side, the 2 o’clock position of the pedicle. The Jamshidi needle is then advanced, via malleting, into the vertebral body. Great care is taken not to pass the medial border of the pedicle on AP fluoroscopy. The needle is advanced to about 25 mm without breaching the medial wall of the pedicle on AP fluoroscopy. Once all the Jamshidi needles are placed in the fashion, lateral fluoroscopy is used to confirm depth and trajectory. The needle is adjusted or advanced as needed, following which guide wires are placed into the vertebral body. Serial dilators are then used over the guide wires followed by a cannulated tap. Afterward, the cannulated tap is removed, as are the dilators. A cannulated pedicle screw with an attached extender is then placed over the guidewire.

**Fig. 5:**
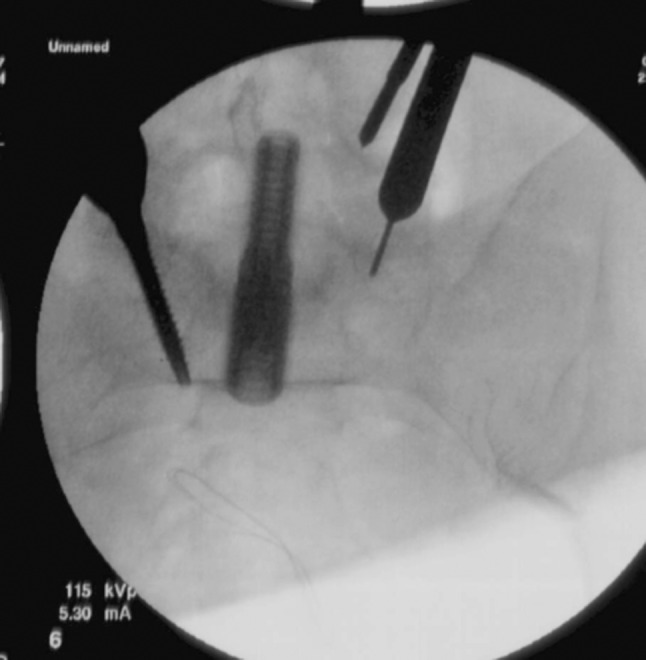
Pelvic inlet view demonstrating tricortical percutaneous S1 pedicle screw placement. Also, note the placement of the AxiaLIF screw (note that this is a different patient than shown in earlier pictures)

For sacral screws, we prefer tricortical screws as per Lehman et al. [17]. A pelvic inlet view is used to confirm tricortical tap and screw placement (Fig. 5). Additionally, on occasion we have found the need to perform S2 or iliac screws. The teardrop view can be performed to place these screws if necessary to extend the fusion to the pelvis [18, 19].

**Fig. 6:**
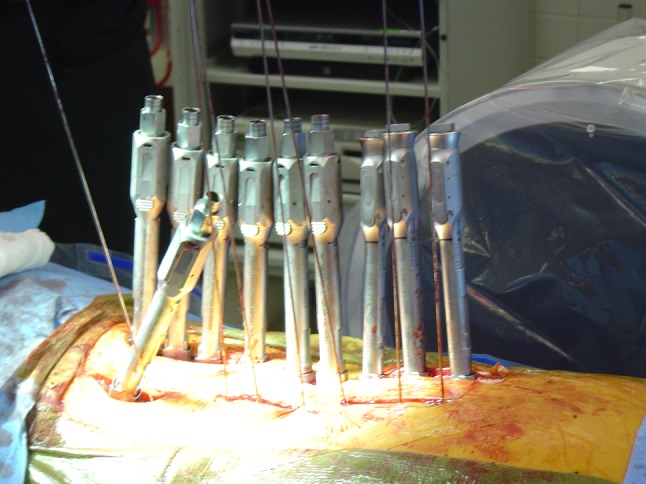
Extenders are lined up in preparation for rod passage (note that this a different patient than shown in earlier pictures)

**Fig. 7:**
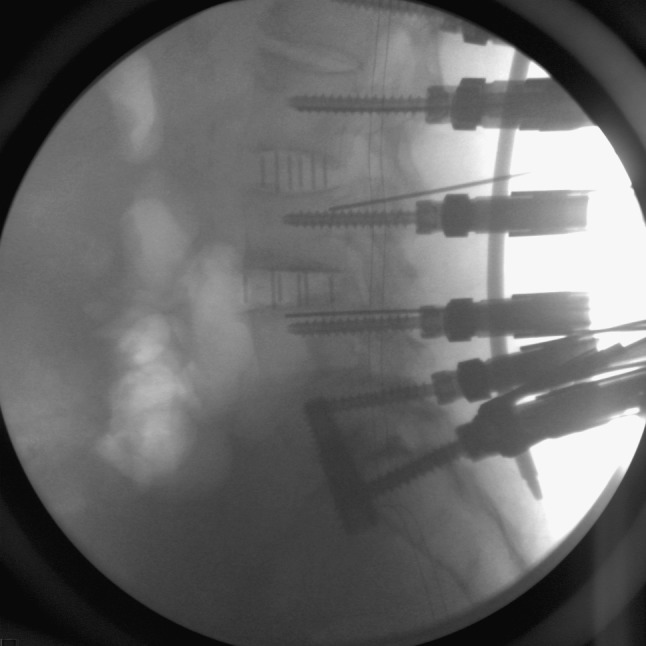
Lateral fluoroscopic view confirming percutaneous rod placement through the extenders of the distal lumbar screws and the sacral screws (note that this a different patient than shown in earlier pictures)

**Fig. 8:**
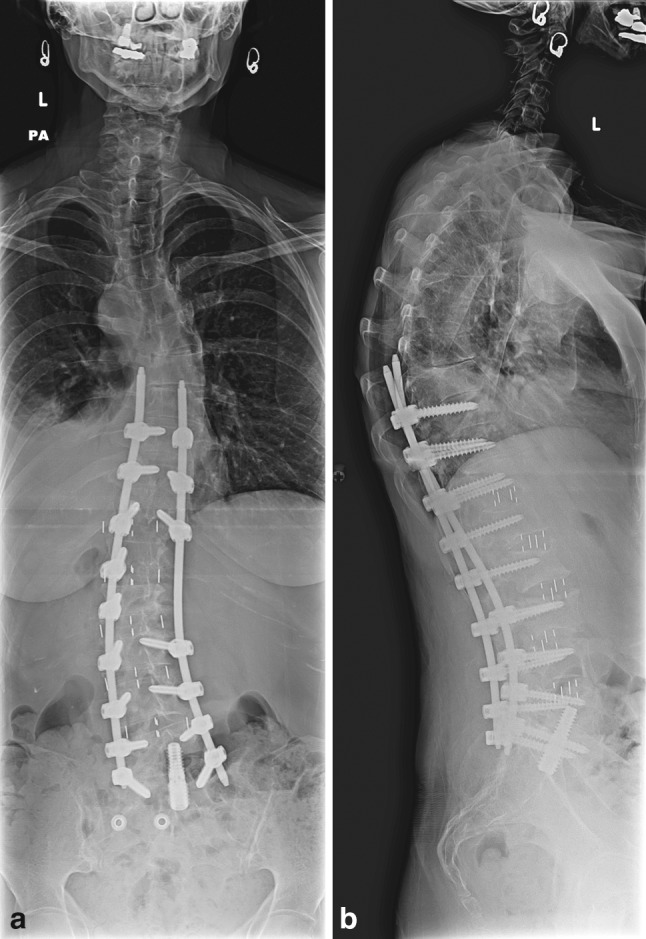
Final 36-inch standing films posttranspsoas discectomy and interbody fusion, L5–S1 transsacral discectomy and interbody fusion and percutaneous pedicle screw and rod placement. The thoracolumbar curve measures 23° and the thoracic curve measures 24°. The majority of the deformity correction was achieved with percutaneous screw and rod placement and not with the transpsoas approach

The cannulated pedicle screws that we have most experience with are the Medtronic CD Horizon Longitude (Medtronic Sofamor Danek, Memphis, TN). These have extenders that are then all lined up (Fig. 6). A measuring device is used to measure the appropriate rod length. An appropriate sized rod is then chosen, loaded onto the introducer and bent to approximate the normal thoracic kyphosis and lumbar lordosis. The rod is then passed with a rod-passing device through a stab incision just rostral to the most proximal pedicle screw, either in the fascia or through the skin. The rod may be contoured in situ with custom instruments as needed. The rod’s presence is confirmed either visually in the extenders and/or with a tester (Fig. 7). The extenders are then sequentially reduced working from caudal to rostral till the rod is reduced into the tulips of the screw. During the reduction maneuver, it is critical to maintain the rod in a strict sagittal orientation. This allows for translation of the apex and reorientation coronally and sagittally. Appropriate compression or distraction maneuvers can be performed as necessary. Derotation also can be performed by manipulating the extenders as reduction is achieved. A vertebral column manipulating device is also available, though we have not found it necessary to achieve derotation. The rod contouring and alignment are key for correction of apical vertical translation. The initial interbody fusion technique allows for a powerful release of the facets in order to correct the deformity more easily with a rod. We have noted, however, that the transpsoas interbody fusion themselves did not result in an adequate deformity rotational correction (Fig. 8).

Once fixation and correction are achieved, facet joint fusions are performed. The lumbosacral facets are always fused bilaterally, but we also perform a facet joint fusion of the thoracolumbar spine especially in cases where interbody fusion is not used. A speculum is used to get down to the facet. The pars and facet joint at each level are then decorticated with a high-speed bur with careful attention paid to packing the local bone back into the facet. Fusion is then achieved with approximately 1–1.5 mg of rhBMP-2 mixed with Grafton putty per pars–facet complex [9].

We have also used neuronavigation for all the above procedures and these techniques can readily be adopted for use with a neuronavigation.

## Outcomes

We previously reported the mid- to long-term outcomes for minimally invasive correction and fusions for adults with scoliosis [9]. A retrospective study of 28 consecutive patients was performed where patients underwent minimally invasive correction and fusion over three or more levels for adult scoliosis greater than 15°. Hospital and office charts were reviewed for clinical data. The mean age of patients in the study was 67.7 years. Mean follow-up time was 22 months. Estimated blood loss for anterior procedures including transpsoas discectomy and interbody fusions was 241 cc, ranging from 20 to 2,000 cc. Estimated blood loss for posterior fusions including L5–S1 transsacral interbody fusion, in some case L4–L5 and L5–S1 transsacral interbody fusion and percutaneous screw fixation and deformity correction, was 231 cc. The mean operating time was 232 min for the anterior procedures and 248 min for the posterior procedures. Mean length of stay in the hospital was 10 days. Preoperative Cobb angle on mean was 22°, ranging from 15° to 62°, which corrected to 7°, ranging from 0° to 22°. All patients maintained correction of their deformity. Solid arthrodesis was confirmed on all patients on plane radiographs. This was further confirmed on 21 patients via CT scan.

In terms of clinical outcomes, the mean preoperative visual analog score was 7.05. Postoperatively, this was 3.03. Mean Oswestry Disability Index preop was 39.13; postop this was 7. Mean preop SF-36 was 57.73 and postop was 61.5. In terms of complications, two patients developed a quadriceps palsy, from which they recovered within 6 months. One patient sustained an acute blood loss of 2,000 cc, developing a retrocapsular renal hematoma, which tamponaded off and required no further treatment other than a blood transfusion. We believe the retractor blade may have injured the renal capsule and a postoperative CT angiogram showed an infarct of the superior pole of the kidney. Another one developed an unrelated cerebellar hemorrhage that required craniotomy.

Recently, we have reported on the 2–5 year results of MIS surgery for spinal deformity [20].

A consecutive cohort of 76 patients who had a minimum follow-up of 2 years was included. Mean age was 64 years (20–84). Mean follow-up was 40 months (26–60) with greater than 3 years follow-up in 43 patients. Patients with one-stage same-day surgery (38) had a mean blood loss of 541 ml and a mean surgical time of 277 min. Patients with two-stage surgery (38) had a mean blood loss of 290 ml and surgical time of 185 min for DLIF and 336 ml and 238 min, respectively, for posterior instrumentation including AxiaLIF. Mean hospital stay was 7.8 days (2–27). The mean preop Cobb angle was 24° (range 6°–61°), which was corrected to 10.4° (range 0.6°–28.8°). The preop coronal balance was 25.5 mm (5.2–85.4 mm), which was corrected to 12.4 mm (0–41 mm). The mean preop sagittal balance was 31.3 mm (−64.8 to151 mm), which was corrected to 14.7 mm (−91.7 to 93.4 mm). The preop lumbar AVT was 23 mm (6.7–57 mm), which was corrected to 11.9 mm (0–40.7 mm). A total of 12 patients had adverse events requiring intervention: 4 patients with pseudarthrosis, 4 with stenosis, 1 requiring screw removal, 1 with osteomyelitis and 2 with wound dehiscence.

We also looked at these strategies for curves greater than 30° and avoided the need for osteotomies in obtaining sagittal and coronal correction in well-selected patients [21]. Forty consecutive patients were identified and reported.

The mean age was 58 years (20–81). Mean follow-up was 28 months (9–58). Patients with one-stage same-day surgery had a mean blood loss of 592 ml and a mean surgical time of 333 min. Patients with two-stage surgery had a mean blood loss of 320 ml and a surgical time of 192 min for DLIF and a mean blood loss of 435 ml and a mean surgical time of 257 min for posterior instrumentation and AxiaLIF. The preop Cobb angle was 41° (30°–74.7°) and corrected to 16.6° (4°–42.8°). The preop coronal balance was 33.09 mm (5.5–143 mm) and corrected to 15 mm (0–31 mm). The preop sagittal balance was 44.3 mm (−47 to 160 mm) and corrected to 1.3 mm (−99 to 88 mm). The preop lumbar AVT was 41.7 mm (11.7–90.4 mm) and corrected to 17.9 mm (2.7–33 mm). Seven patients had adverse events requiring intervention: three with L5–S1 pseudoarthrosis, one with stenosis and radiculopathy, one with delayed onset adjacent osteomyelitis, one with sacral wound dehiscence and one with proximal screw prominence.

Outside of these series, we also had two cases of misplaced pedicle screws that needed revision.

### Evidence in the literature supporting a similar approach

Similar outcomes were reported by Wang and Mummaneni [22], who reviewed the transpsoas approach for deformity correction. In their series, they performed minimally invasive transforaminal lumbar interbody fusion at L5–S1 rather than AxiaLIF fusion. In their series, a mean blood loss of 477 cc was noted with a mean operative time of 401 min. They had a mean follow-up of 13.4 months. Mean preoperative Cobb angle was 31.4°, which was reduced to 11.5°. The authors conclude that these technologies remain a promising method of reducing surgical morbidity and correction of spinal deformity.

Similarly, Dakwar et al. [23] reported outcomes on 25 patients who underwent the transpsoas approach for discectomy and interbody fusion for adults with degenerative deformity. With regard to posterior instrumentation, a variety of stabilization techniques were used including lateral plates and open and percutaneously placed pedicle screws. They noted mean blood loss to be 53 cc per segment fused. They also noticed a mean improvement of 5.7 points on the VAS scores, and a 23.7 % improvement in ODIs. Complications in their series included transient postoperative anterior thigh numbness, rhabdomyolysis and hardware failure.

Tormenti et al. [24] reported outcomes in eight patients with MIS transpsoas approach combined with open posterior pedicle screws. They noted a mean Cobb angle correction of 38.5° to 10°. They noted a thigh dysesthesia and motor radiculopathy to be common complications. They also noted a single case of intraoperative bowel injury.

Karikari et al. [25] reported on 22 patients who underwent extreme lateral interbody fusion for isolated thoracic and thoracolumbar spinal disease. Eleven patients had degenerative scoliosis. In the subset of patients treated for degenerative scoliosis, the mean preoperative and postoperative coronal Cobb angles were 22 and 14, respectively. They noted no major neurovascular operative complications.

Issacs et al. [26] reported perioperative outcomes and complications of a prospective multicenter of the extreme lateral interbody fusion procedure in adult degenerative scoliosis. A total of 107 patients underwent XLIF procedure. In terms of additional fixation, 75.7 % used posterior pedicle screws (64.2 % placed using minimal access surgical techniques; 35.8 % using standard open techniques) and 5.6 % used lateral fixation in combination with the XLIF. Mean operative time was 177.9 min (range 43–458 min) per XLIF surgery and 57.9 min per interbody fusion level. Almost two-thirds (62.5 %) of patients had a recorded EBL of <100 mL, and only nine patients (8.4 %) had >300 mL EBL. The average length of hospital stay was 2.9 days for unstaged procedures, 8.1 day for staged procedures and 3.8 days overall.

Thirty-three percent of patients had some lower extremity weakness postop and 6.5 % of patients had weakness that did not resolve by 6 months. Overall, the authors reported a major complication rate of 12.1 % and a minor complication rate of 15.9 %. Notably, the authors reported that the incidence of having any complication was significantly higher in those with open posterior fixation (27.9 %) than those with percutaneous posterior fixation (15.4 %) and that the incidence of major complications was significantly higher (20.7 vs. 5.8 %, *P* = 0.0405) in those undergoing open instrumentation placement. The authors concluded that the transpsoas minimally invasive approach to anterior column reconstruction resulted in reduced blood loss, shorter hospital stays, and less infections, transfusions, early reoperations and perioperative complications than historically reported for traditional open procedures in the treatment of adult scoliosis. They also noted that morbidity/complication rate increased with increased levels of surgery and with the use of open posterior instrumentation.

Of note, all of these numbers compare favorably to patients undergoing more traditional open procedures. Cho et al. [27] reported on a series of patients undergoing PLIF for lumbar degenerative scoliosis. Overall, their complication rate was 68 %, with 30 % having early perioperative complications and 38 % having late complications. Blood loss was a very significant risk factor for early postoperative complications. Their mean blood loss was 2.1 L with an average hospital stay of 20 days. Similarly, Wu et al. [28] reported on 26 patients who underwent PLIF procedure for degenerative scoliosis. They noted a mean blood loss of 1.7 L with an average hospital stay of 11.7, plus or minus 8.3 days.

Bono and Lee reviewed 78 articles regarding outcomes of spinal fusion for lumbar degenerative disc disorders [29]. They reported overall good to excellent outcomes of 82 % of patients undergoing surgery for lumbar degenerative scoliosis. Nevertheless, they noted a pooled complication rate of 55 %. Clearly, based on these numbers, early results of minimally invasive deformity correction and fusion are comparable compared to open surgery.

## Conclusions

Minimally invasive deformity correction and fusion represents a promising method of correction of spinal deformity. Early results are similar to more traditional techniques. Percutaneous screw and rod placement requires excellent intraoperative imaging. Rod bend and placement are critical for final deformity correction. Meticulous surgical technique and patient selection are necessary to avoid complications and obtain good surgical results.
